# Supplementary material for: Developmental timing-dependent organization of synaptic connections between mossy fibers and granule cells in the cerebellum
Source: Commun Biol. 2023 Apr 24;6:446. doi: 10.1038/s42003-023-04825-y (PMC10125988; doi:10.1038/s42003-023-04825-y)
Supplement: Supplementary file 2 — Supplementary Information [file 42003_2023_4825_MOESM2_ESM.pdf]

Supplementary Information

Developmental timing-dependent organization of synaptic connections between mossy fibers and granule cells in the cerebellum

Taegon Kim, Heeyoun Park, Keiko Tanaka-Yamamoto, and Yukio Yamamoto

Correspondence to Yukio Yamamoto (yukio.kist@gmail.com) or Keiko Tanaka-Yamamoto (keikoyamat@gmail.com)

This PDF file includes Supplementary Figs. 1 to 9

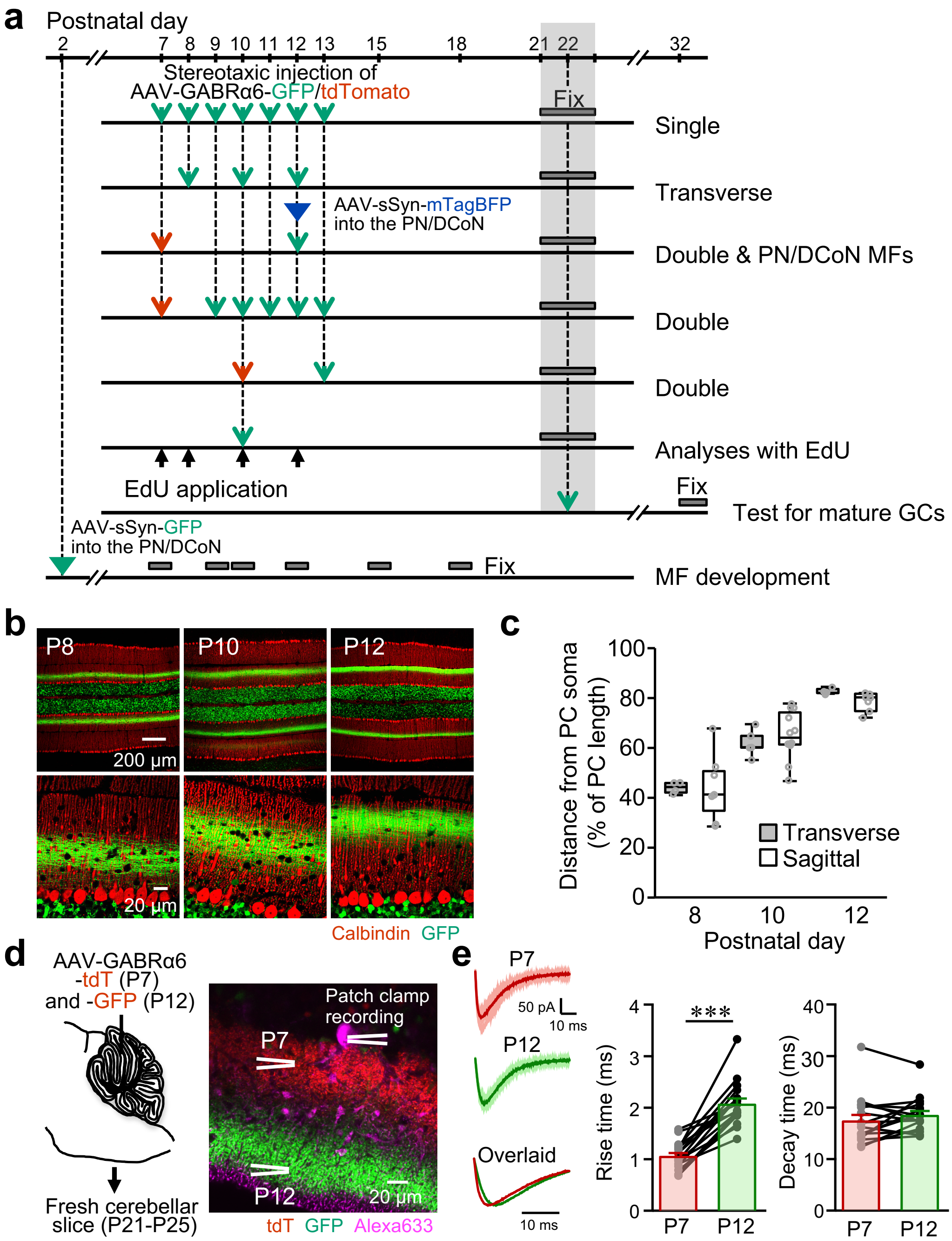

Supplementary Fig. 1: Experimental time course and locations of PFs labeled by AAV-GABRα6-GFP injection at different postnatal days.
(**a**) The diagram showing time of injection of AAV-GABRα6-GFP (green arrows) or -tdT (red arrows), AAV-sSyn-GFP (green triangle) or -mTagBFP (blue triangle), intraperitoneal application of EdU (black arrows), and the fixation (gray horizontal bars) followed by imaging analyses. (**b**) Confocal images of cerebellar transverse slices labeled by AAV-GABRα6-GFP (green) injection at indicated postnatal days. Slices are stained with a calbindin antibody (red). (**c**) Distance between the labeled PF bundles and PC somas. Distances measured in transverse slice images (*N* = 4-5 mice, 13 mice in total, 3 images per mouse) are compared with those in sagittal slice images shown in Fig. 1b. Data are presented as boxplots. (**d**) Schematic drawing showing time of AAV injection and patch clamp experiments (left), and a representative image of a fresh sagittal slice (right). (**e**) Rise time and decay time of PF-EPSCs elicited by stimulating PFs that were labeled by either P7 (red) or P12 (green) injection. ***$p < 10^{-6}$ (rise time), $p = 0.202$ (decay time), paired Student's *t*-test (*n* = 16 slices). Data are presented as mean ± s.e.m. and circles represent individual data points.

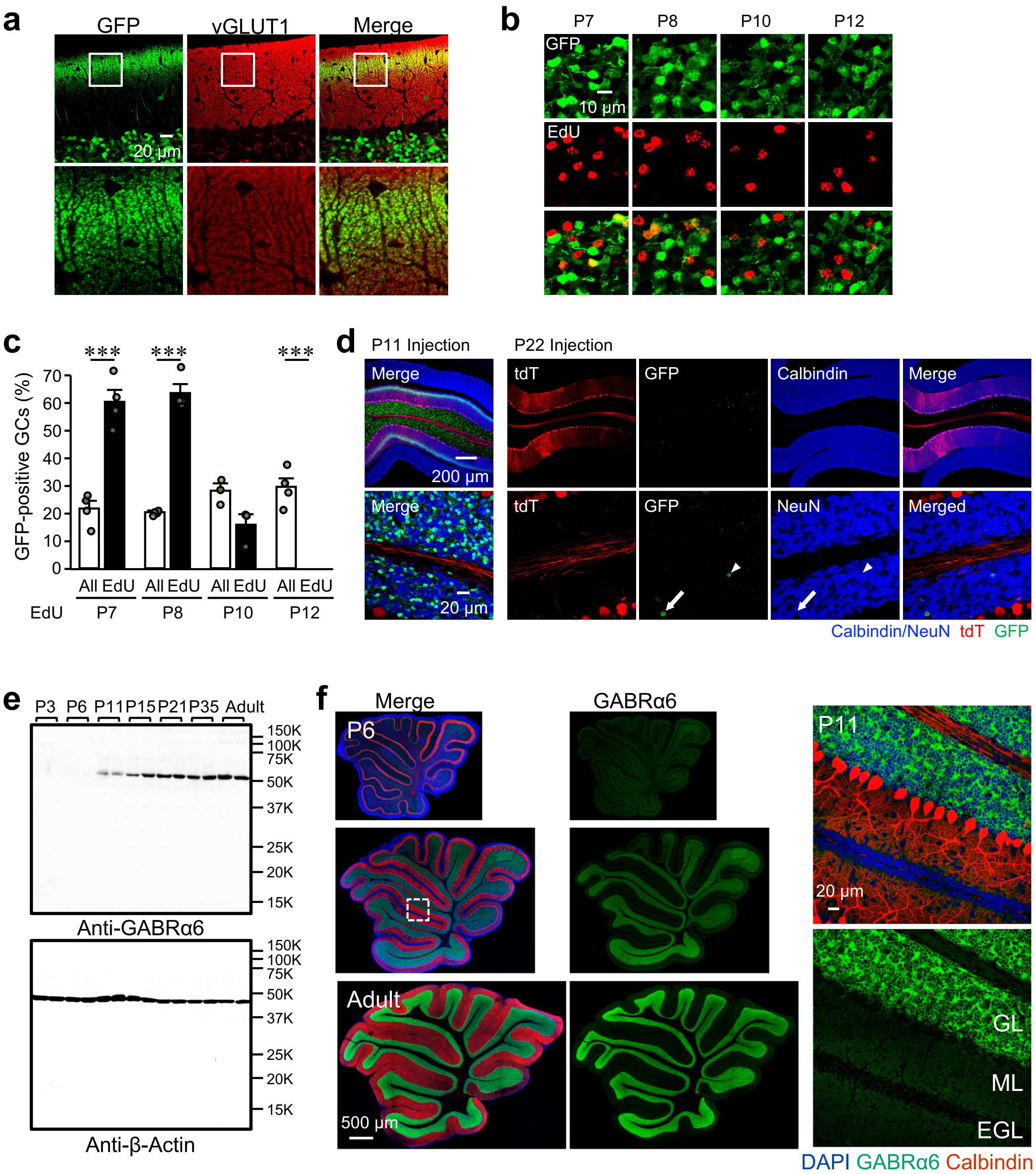

Supplementary Fig. 2: Expression of molecules triggered by AAV-GABRα6 and expression patterns of endogenous GABRα6.

(**a**) Confocal images of cerebellar sagittal slices stained with a vGLUT1 antibody (red). The area in the white square is magnified in the panel underneath. Note that GFP puncta (green) are well overlapped with vGLUT1 staining in the ML. (**b**) Representative images of EdU (red) and GFP (green) signals, after EdU (P7, P8, P10, or P12) administration together with AAV-GABRα6-GFP injection at P10. (**c**) Percentages of GFP-positive GCs in all GCs or in EdU-positive GCs (N = 3–4 mice for each timing of EdU administration, for comparison between all and EdU at each timing of EdU administration; $^{***}p = 1.19 \times 10^{-8}$ (P7), $^{***}p = 1.6 \times 10^{-9}$ (P8), $p = 0.063$ (P10), $^{***}p = 1.15 \times 10^{-6}$ (P12), two-way ANOVA followed by the Bonferroni test). Data are presented as mean ± s.e.m. and circles represent individual data points. (**d**) Confocal images of cerebellar slices obtained from mice injected with AAV-GABRα6-GFP (green) and AAV-CaMKIIα-tdT (red) at P11 (left) or P22 (right). Slices were stained (blue)

with an antibody against calbindin (top) or NeuN (bottom, a marker of GC somas). The arrowhead indicates a NeuN and GFP double-positive cell, whereas the arrow indicates a NeuN-negative and GFP-positive cell. (**e** and **f**) Western blot (**e**) and immunohistochemical (**f**) analyses to see the expression of endogenous GABRα6 protein in developing and adult mice. For the western blot, a β-Actin antibody was also used. Uncropped and unedited blot images are provided in Supplementary Fig. 9. For the immunohistochemistry, slices were stained with a calbindin antibody and DAPI. In (**f**), the area within a white square in the image of P11 is magnified on the right.

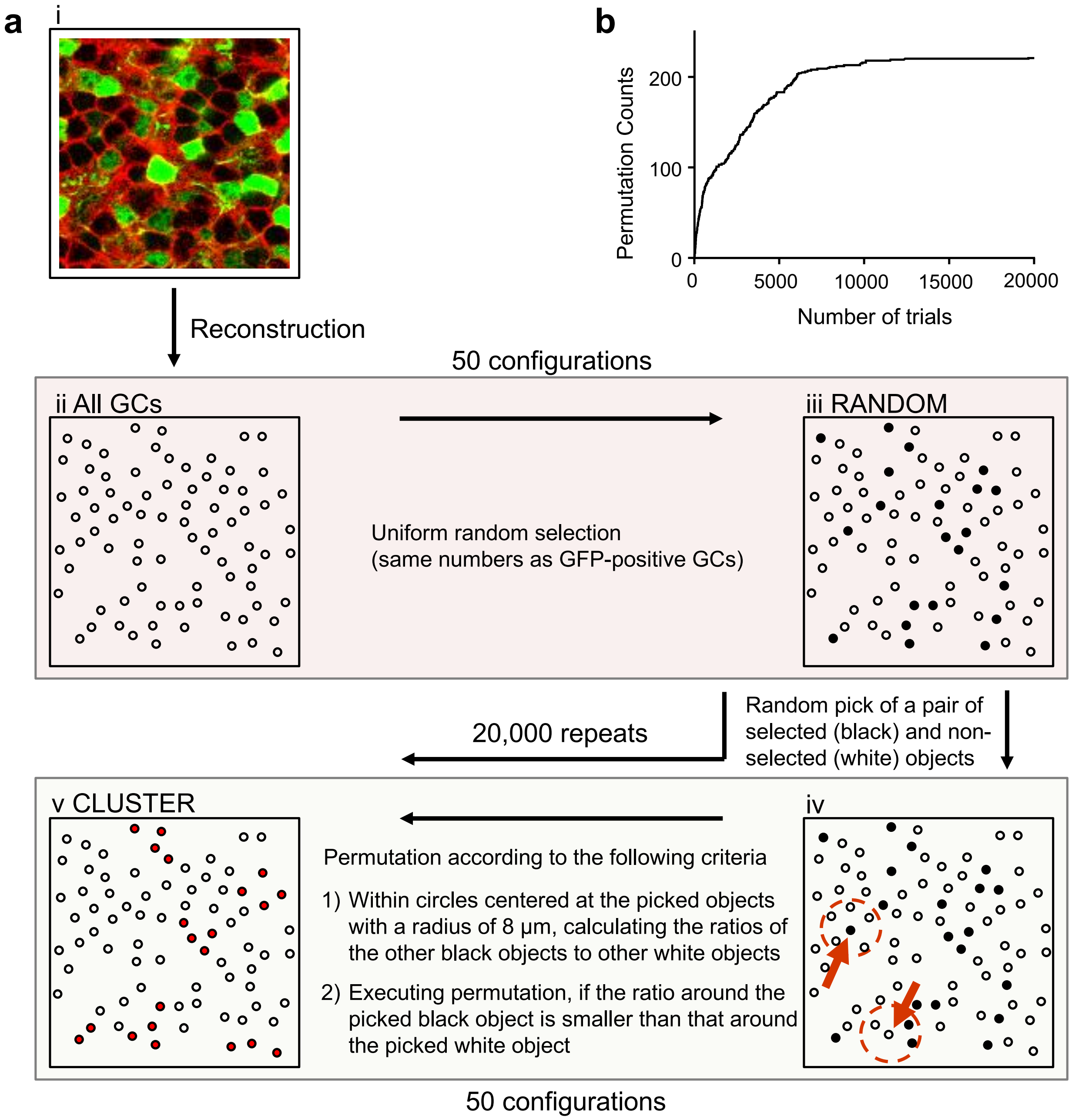

Supplementary Fig. 3: Generation of the random or clustered configurations of GC somas.

(**a**) Flow chart of how to create random and clustered configurations. Experimentally obtained positions of all GC somas (i) were computationally reconstructed (ii), and an equivalent number of GCs as GFP-positive GCs was randomly selected (iii, sGC, black circles). The distribution of sGCs was used as random configurations, and 50 examples were obtained from single original image. To create clustered configurations, a pair of one sGC and one non-sGC was picked, and ratios of other sGC to other non-sGC numbers were compared (iv). If the ratio around the picked sGC was smaller than the ratio around the picked non-sGC, the position of this pair of GCs was permutated. This process of pair picking and permutation was repeated 20,000 times to convert from a random configuration to a clustered configuration (v). (**b**) An example of the relationship between number of trials and permutation counts. The permutation counts were approximately stabilized when the process was repeated 10,000–20,000 times.

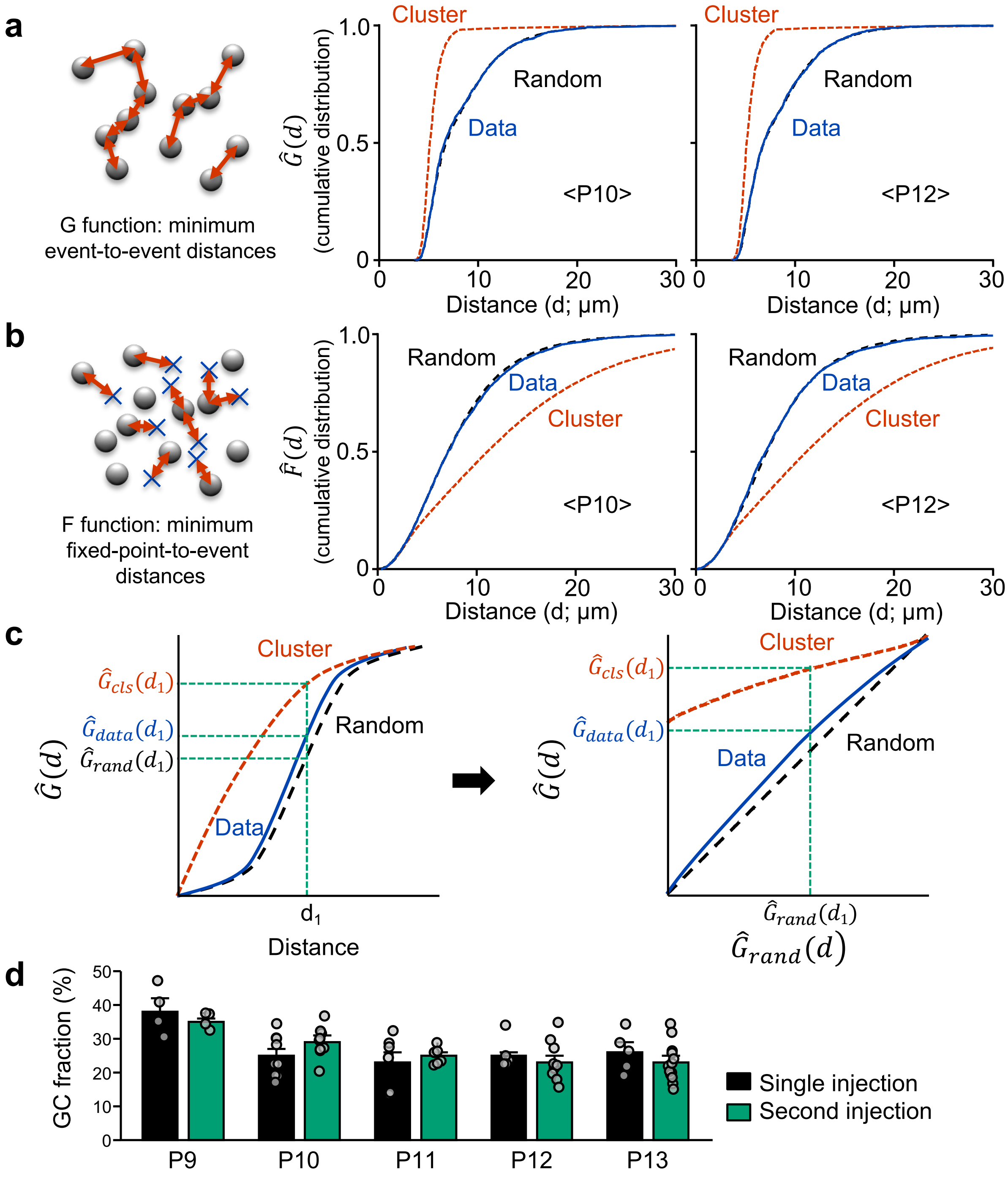

Supplementary Fig. 4: Distributions of GFP-positive GC somas.
(**a** and **b**) Schematic explanations (left) and examples (right) of $\hat{G}$ (**a**) and $\hat{F}$ functions (**b**). The data used for examples were obtained from sagittal slice images of P10 and P12 injection. Experimental results are shown in blue solid lines, and analyzed data from computationally created random (black dotted lines) or clustered (red dotted lines) configurations are overlaid. (**c**) Schematic illustration explaining how $\hat{G}(d)$ or $\hat{F}(d)$ was replotted against $\hat{G}_{rand}(d)$ or $\hat{F}_{rand}(d)$. (**d**) Confirmation of similar expression percentages in GCs achieved by the single injection and the second time of double injection of AAV-GABRα6-GFP on the indicated postnatal days. AAV-GABRα6-tdT was injected before the second time of double injection. For the comparison, percentages of GFP-positive GCs shown in Fig. 4c are also used in this figure. p = 0.686 (P9), p = 0.156 (P10), p = 0.937 (P11), p = 0.277 (P12), p = 0.387 (P13), Mann–Whitney test (N = 4–13 mice each, 74 mice in total). Data are presented as mean ± s.e.m. and circles represent individual data points.

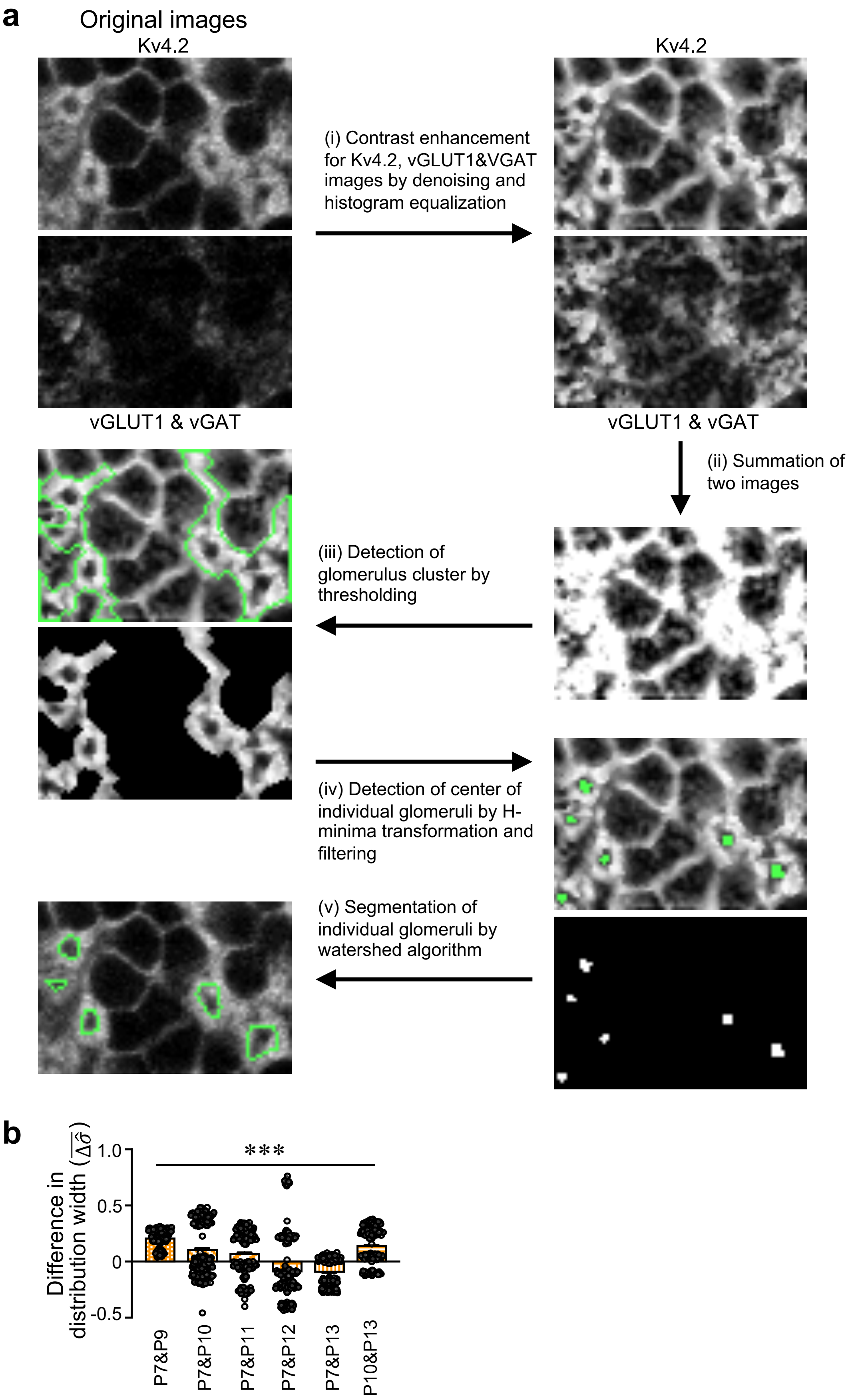

Supplementary Fig. 5: Procedures of individual glomerulus segmentation.
(**a**) Flow chart of how to achieve segmentation of individual glomeruli in images of staining with Kv4.2, vGLUT1 and vGAT antibodies. The image contrast was enhanced by denoising and histogram equalization (i), and the summed images (ii) were used to detect glomerulus

areas (iii). The center of individual glomeruli was then detected in Kv4.2 images within the whole glomerulus areas (iv). The detected centers were used as markers to filter the segmented regions by watershed algorithm (v). (**b**) Individual data points for comparisons of distribution broadness between experimental results and shuffled data. Averaged data shown in Fig. 3e are also included.

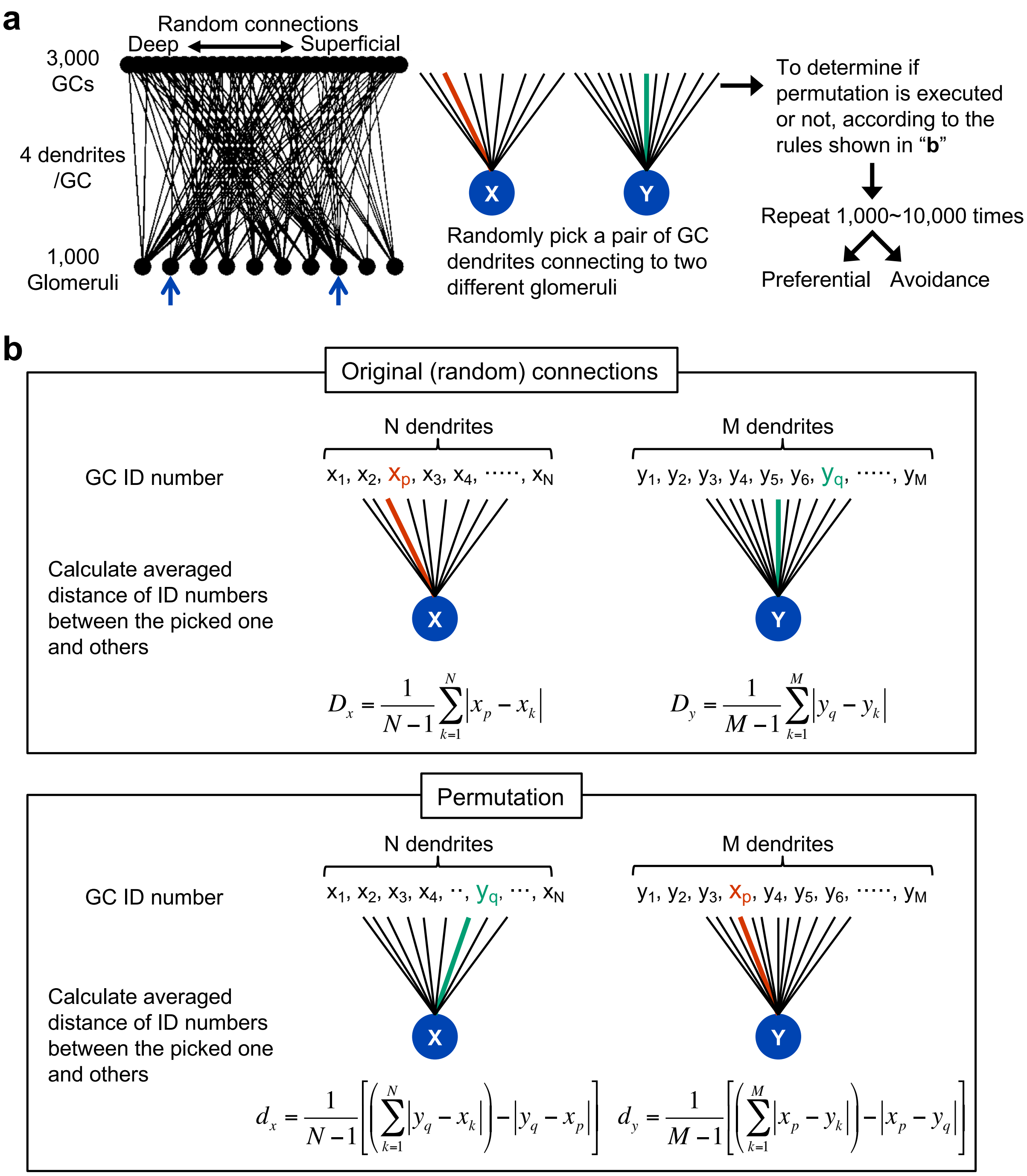

Counting	Connections	Permutation	Features
$D_x + D_y < d_x + d_y$	Preferential	–	Narrower range of IDs
	Avoidance	Execute	To make wider range of IDs
$D_x + D_y > d_x + d_y$	Preferential	Execute	To gather narrower range of IDs
	Avoidance	–	Wider range of IDs

Supplementary Fig. 6: Computational network model of preferential and avoidance connections.
(**a**) Flow chart to create preferential and avoidance connections from the random connection. The network model consisted of 3,000 GCs and 1,000 glomeruli, latter of which can be also considered as MF terminals. A randomly picked pair of dendrites connecting to different glomeruli was either permutated or not, according to the rules described in (**b**). This process

was repeated 1,000–10,000 times. (**b**) Rules of permutation. The averaged distances between the ID numbers of picked GC dendrites and those of others in the glomeruli were calculated, and the averaged distances were compared between original case ($D_x + D_y$) and permutated case ($d_x + d_y$). The permutation was executed, if $D_x + D_y$ was smaller than $d_x + d_y$ in case of creating avoidance connections, or if $D_x + D_y$ was larger than $d_x + d_y$ in case of creating preferential connections, because avoidance connections must have larger distances, while preferential connections must have smaller distances.

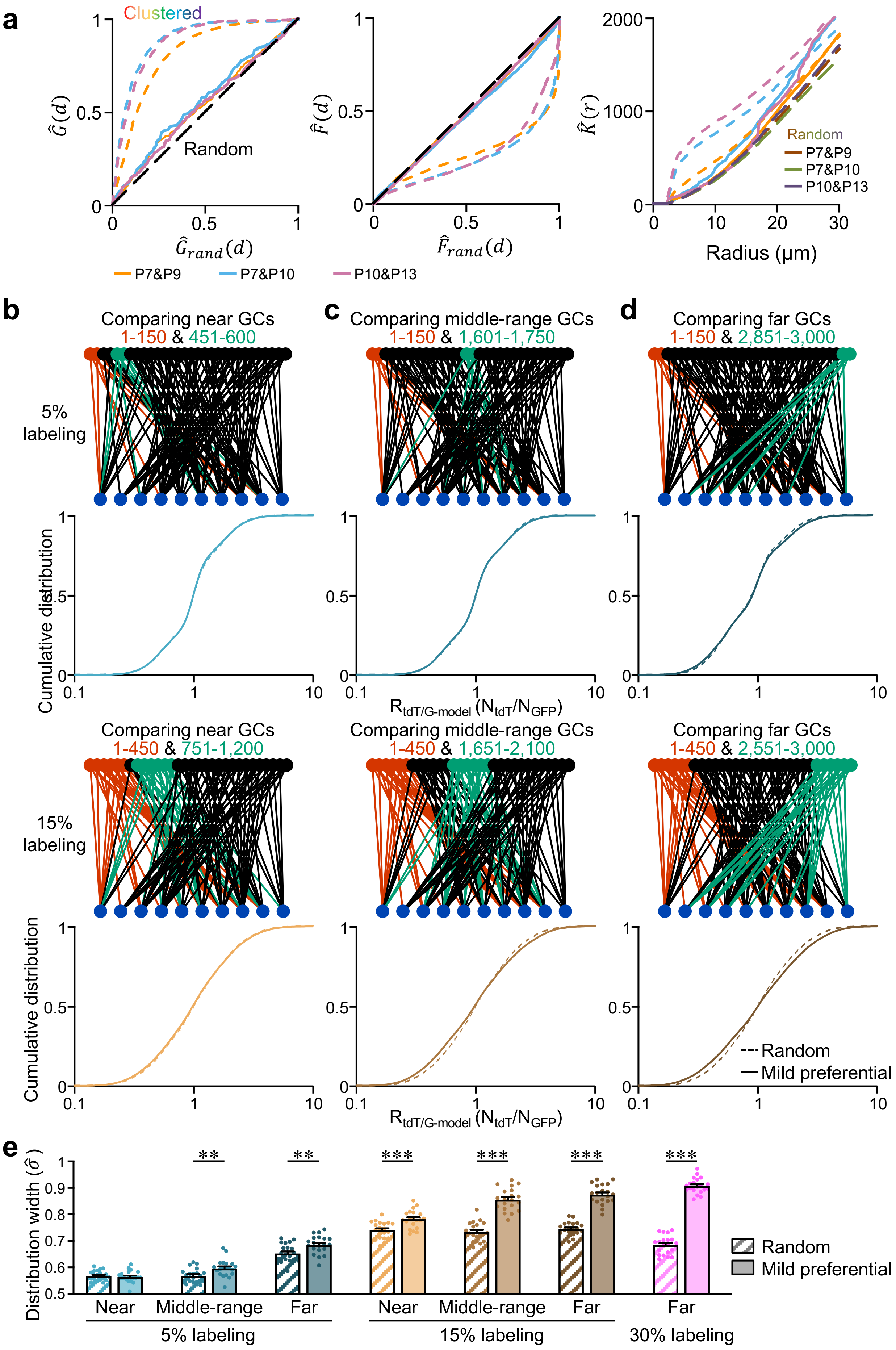
a
Clustered
Random
P7&P9
P7&P10
P10&P13
Radius (μm)
b
Comparing near GCs
1-150 & 451-600
5% labeling
Cumulative distribution
c
Comparing middle-range GCs
1-150 & 1,601-1,750
d
Comparing far GCs
1-150 & 2,851-3,000
$R_{tdT/G\text{-}model}$ (N_{tdT}/N_{GFP})
Comparing near GCs
1-450 & 751-1,200
15% labeling
Comparing middle-range GCs
1-450 & 1,651-2,100
Comparing far GCs
1-450 & 2,551-3,000
Random
Mild preferential
e
Distribution width ($\hat{\sigma}$)
Near
Middle-range
Far
5% labeling
15% labeling
30% labeling
Random
Mild preferential

Supplementary Fig. 7: Analyses with small numbers of GCs labeled.
(**a**) The analyses of distributions of tdT/GFP double-labeled GCs in sagittal planes by the $\widehat{G}$ (left), $\widehat{F}$ (middle), or $\widehat{K}$ functions (right) (N = 4–6 mice, 15 mice in total, 2 images per mouse), when two groups of GCs were labeled by double injection at P7&P9, P7&P10 or P10&P13. Black (for $\widehat{K}$ functions, dark colored) and colored dotted lines show the results obtained from computationally created random and clustered configurations, respectively. Solid lines show the experimental results. (**b-e**) Comparisons of cumulative distributions (**b-d**) or distribution broadness (**e**) of $R_{tdT/G\text{-}model}$ between random (dotted lines) and mild preferential (solid lines) connection models, under assumption of a group of GCs consisting of 5% (top in **b-d**) or 15% (bottom in **b-d**) GCs with a consecutive ID. As shown in diagrams, two groups of GCs located at near (**b**), middle (**c**), or far (**d**) distance were labeled by tdT and GFP. For the direct comparison, distribution broadness obtained from data shown in Fig. 5c (top-left panel, far comparison) is also presented in **e** (30%, far). The x-axes of **b-d** are in log scale. p = 0.3759 (5%, near), **p = 0.0053 (5%, middle-range), **p = 0.0014 (5%, far), ***p = 0.0005 (15%, near), ***$p = 1.5 \times 10^{-9}$ (15%, middle-range), ***$p < 10^{-10}$ (15%, far), ***$p < 10^{-10}$ (30%, far), paired Student's *t*-test. In **e**, data are presented as mean ± s.e.m. and circles represent individual data points.

a Numerical sequences to show type of GCs connecting to individual MF terminals: {D-GC, M-GC, S-GC}

<Examples>

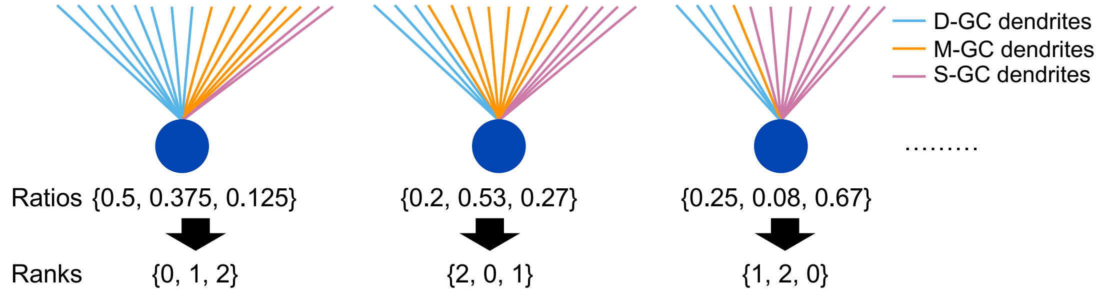

b Dominancy-dependent categorization: ratio sequence

Arrange sequences in descending order of ________	D-GC ratios	M-GC ratios	S-GC ratios
Arrange sequences	{**1**, 0, 0}<br>•<br>•<br>•<br>{**0.9**, 0.02, 0.08}<br>•<br>•<br>•<br>{**0.5**, 0.375, 0.125}<br>•<br>•<br>•	{0, **1**, 0}<br>•<br>•<br>•<br>{0.07, **0.9**, 0.03}<br>•<br>•<br>•<br>{0.2, **0.53**, 0.27}<br>•<br>•<br>•	{0, 0, **1**}<br>•<br>•<br>•<br>{0.01, 0.09, **0.9**}<br>•<br>•<br>{0.25, 0.08, **0.67**}<br>•<br>•<br>•<br>•
Top 33% glomeruli	D-GC-dominant MF terminals	M-GC-dominant MF terminals	S-GC-dominant MF terminals

c Order-dependent categorization: rank sequence

Select sequences of ________	{0, 1, 2}	{0, 2, 1}	{2, 0, 1}	{1, 0, 2}	{1, 2, 0}	{2, 1, 0}
Glomeruli with all selected sequences	D>M>S MF terminals	D>S>M MF terminals	M>S>D MF terminals	M>D>S MF terminals	S>D>M MF terminals	S>M>D MF terminals

Supplementary Fig. 8: Dominancy- and order-dependent categorization of MF terminals.

(**a**) Numerical sequences are made to show ratios or ranks of dendrite numbers of D-GCs, M-GCs, and S-GCs among all GC dendrites connecting to individual MF terminals. The rank 0 means the most abundant GC dendrites, and the rank 2 means the fewest GC dendrites. Three examples are shown. (**b**) The ratio sequences were arranged in descending order of D-GC, M-GC, or S-GC ratios, and top 33% MF terminals were considered as D-GC-, M-GC-, or S-GC-dominant MF terminals, respectively. (**c**) Individual MF terminals were also categorized into six populations according to rank sequences.

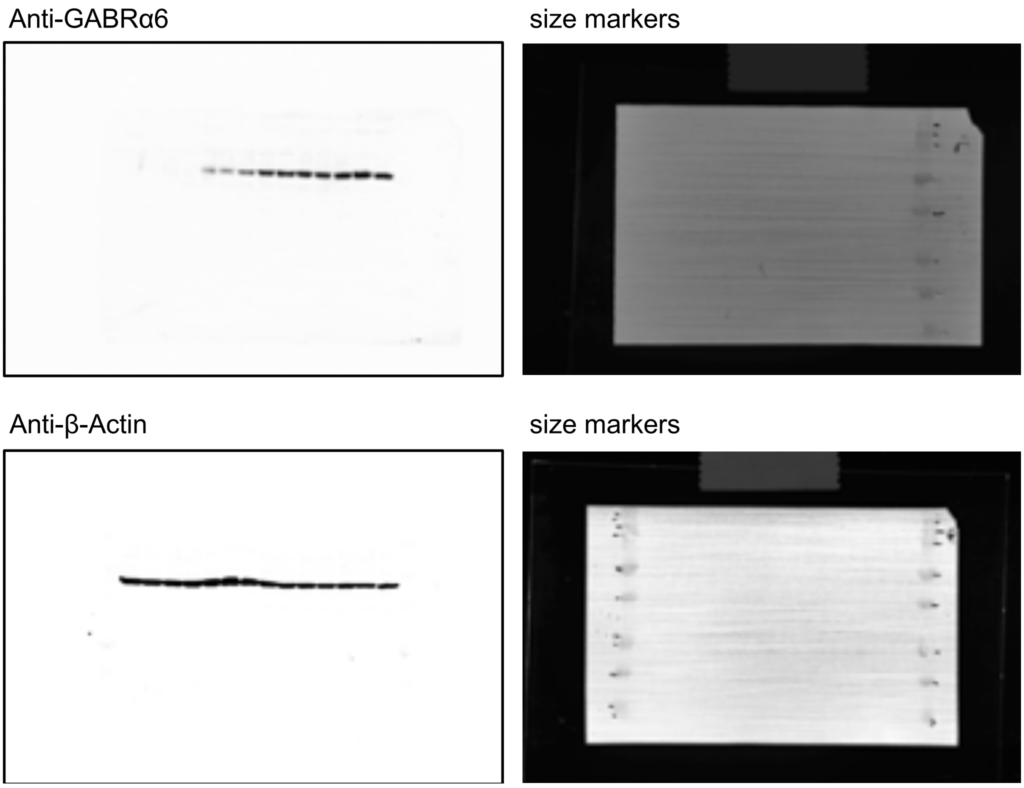

Supplementary Fig. 9: Uncropped and unedited blot images of Supplementary Fig. 2e
