# Supplementary material for: Developmental timing-dependent organization of synaptic connections between mossy fibers and granule cells in the cerebellum
Source: Commun Biol. 2023 Apr 24;6:446. doi: 10.1038/s42003-023-04825-y (PMC10125988; doi:10.1038/s42003-023-04825-y)
Supplement: Supplementary file 3 — Description of Additional Supplementary Files [file 42003_2023_4825_MOESM3_ESM.pdf]

Description of Additional Supplementary Files

File name: Supplementary Data 1

Description: Experimentally obtained numerical data
